# Sustainable Cyanobacterial Bloom Control: Inhibitory Effects of Nano Zero-Valent Iron on *Microcystis aeruginosa* and Metabolic Disruption

**DOI:** 10.3390/toxics13110915

**Published:** 2025-10-24

**Authors:** Guoming Zeng, Zilong Ma, Xiaoling Lei, Yong Xiao, Da Sun, Yuanyuan Huang

**Affiliations:** 1 Chongqing Academy of Science and Technology, Chongqing 401123, China; 2 School of Civil Engineering, Chongqing Jiaotong University, Chongqing 400074, China; 3 School of Civil and Hydraulic Engineering, Chongqing University of Science and Technology, Chongqing 401331, China; 4 Zhejiang Provincial Key Laboratory of Water Ecological Environment Treatment and Resource Protection, College of Life and Environmental Science, Wenzhou University, Wenzhou 325035, China; 5 Institute of Life Sciences and Biomedical Collaborative Innovation Center of Zhejiang Province, Wenzhou University, Wenzhou 325035, China

**Keywords:** nZVI, cyanobacterial blooms, *Microcystis aeruginosa*, inhibition mechanism, metabolomics

## Abstract

The bloom of cyanobacteria has severely disrupted ecological balances, posing significant risks to human health and safety. However, there is currently a lack of environmentally friendly methods that can sustainably suppress these blooms over the long term. This study integrates untargeted metabolomics, Fourier-transform infrared spectroscopy (FTIR), and scanning electron microscopy (SEM) to systematically characterize the responses of *Microcystis aeruginosa* to nano zero-valent iron (nZVI). Exposure to nZVI reprograms lipid and amino acid metabolism, coincides with the suppression of protein biosynthesis, and perturbs central pathways—including the tricarboxylic acid (TCA) cycle, photosynthesis, and carbohydrate metabolism—leading to disruptions in energy balance and metabolic homeostasis. FTIR and SEM provide complementary evidence of membrane compromise, with attenuation of -OH, -C-H, and C=O functional group signals, abnormal cell morphology, and progressive oxidative injury culminating in cell lysis and solute leakage. Together, these results support the inhibitory effect of nZVI on *M. aeruginosa* and provide insights to guide metabolomics studies of *M. aeruginosa* using nZVI.

## 1. Introduction

Water is the essential foundation for human society’s survival and development, and its quality affects the balance of the ecological environment and the progression of socio-economic growth. When urban domestic sewage and industrial–agricultural wastewater are discharged into rivers, excess organic matter and nutrients such as nitrogen and phosphorus lead to excessive algae growth, thereby diminishing the water body’s self-purification capability and triggering eutrophication. In particular, cyanobacterial blooms dominated by *Microcystis aeruginosa* (*M. aeruginosa*) have become a significant threat to global freshwater ecosystems and a major issue in water environment pollution [1]. Hence, it is paramount to adopt effective strategies to control cyanobacterial cells without adversely impacting the aquatic ecosystem.

With the rapid advancement of nanotechnology, the application of nanomaterials in environmental pollution control has become increasingly widespread, particularly in the significant progress made in inhibiting algae growth. Metal and metal oxide nanoparticles (such as silver, titanium dioxide, and zinc oxide nanoparticles) effectively inhibit algae by disrupting algae cell membranes and generating ROS [2,3]. Simultaneously, carbon-based nanomaterials (such as graphene and carbon nanotubes) achieve algae inhibition through light-blocking effects and physical interactions [4]. However, these technologies exhibit certain limitations in practical applications, presenting a dilemma [5]. For instance, many nanomaterials face challenges related to ecological safety, cost, and large-scale production, and they pose potential toxicity to non-target organisms. nZVI, as an emerging material, demonstrates significant potential in environmental remediation, particularly in addressing the issue of algae blooms. It exhibits selective toxicity towards cyanobacteria, while its impact on other aquatic organisms remains relatively minimal [6]. This not only mitigates potential adverse effects on ecosystems but also enhances its feasibility for large-scale application.

Marsalek et al. [6] discovered that nZVI induces oxidative stress through its reaction with water. The active transport of ferrous ions leads to the rapid disintegration of the cell membrane, while the subsequent formation of substantial ferric hydroxide precipitates further removes the cyanobacteria. Similarly, Su et al. [7] found that aged nZVI remains effective in removing the growth of cyanobacteria, likely due to Fe(II) ions and iron oxides generating free radicals that result in cell lysis. Jiang et al. [8] point out that nZVI may also impair the absorption of light by algae cells through a shading effect, thereby damaging organelles and cell membranes. These studies suggest that nZVI can induce Fenton-like reactions and shading effects, thereby acting on intracellular substances to inhibit the growth of cyanobacteria. However, most current research focuses on using nZVI technology to remove cyanobacteria that have already proliferated extensively. Although this method is effective in certain cases, it typically requires a significant amount of nZVI, which is not only costly but often less efficient than traditional adsorption and precipitation methods. Additionally, the issue of nZVI recovery remains. Recent evidence suggests that nZVI can affect or inhibit cellular metabolism [9,10], indicating its potential to suppress cyanobacterial growth. This implies that using nZVI as a persistent growth inhibitor before a cyanobacterial bloom, rather than as a removal agent afterward, could be a forward-thinking, environmentally friendly, and cost-effective strategy for controlling cyanobacterial outbreaks. However, its efficacy and mechanisms in inhibiting cyanobacterial growth are not yet well understood.

In this context, this study investigates the inhibitory effects of nanoscale zero-valent iron (nZVI) on *M. aeruginosa*. Specifically, untargeted metabolomics was employed to profile nZVI-induced alterations in differential metabolites and metabolic pathways in *M. aeruginosa*, and, together with scanning electron microscopy (SEM) and Fourier-transform infrared spectroscopy (FTIR), to elucidate the mechanisms by which nZVI inhibits *M. aeruginosa*.

## 2. Experimental Methods

### 2.1. Strain Cultivation and Reagents

*M. aeruginosa* (FACHB-913) was procured from the Institute of Aquatic Biology, Chinese Academy of Sciences, and cultured in BG11 medium under controlled conditions in a light incubator. The culture parameters were maintained at (25 ± 1) °C, with a light intensity of 46 μmol·m^−2^·s^−1^, a 12 h:12 h light-dark cycle, daily shaking (1–2 times), and aeration. Ferrous sulfate heptahydrate (FeSO_4_·7H_2_O) (Chengdu Kelong Chemical Co., Ltd., Chengdu, China), anhydrous ethanol (C_2_H_5_OH) (Chengdu Kelong Chemical Co., Ltd., Chengdu, China), sodium borohydride (NaBH_4_) (Tianjin Kemiou Chemical Reagent Co., Ltd., Tianjin, China), sodium hydroxide (NaOH) (Chengdu Kelong Chemical Co., Ltd., Chengdu, China), hydrochloric acid (HCl) (Chengdu Kelong Chemical Co., Ltd., Chengdu, China), and glacial acetic acid (Chengdu Kelong Chemical Co., Ltd., Chengdu, China) were of analytical grade and used without further purification. Ultrapure water (18.25 MΩ·cm) was utilized for all experiments.

### 2.2. Preparation of nZVI

The following chemical reaction [11] was used to synthesize nZVI from sodium borohydride and ferrous sulfate solution (see Section S1 for the detailed preparation process).

2Fe^2+^ + BH4^−^ + 2H_2_O = 2Fe^0^ + BO_2_^−^ + 2H_2_ + 4H^+^ (1)

### 2.3. Cell Membrane Integrity Assay and Characterization of Algal Cells

Changes in algal cell functional groups before and after nZVI treatment were analyzed using Fourier transform infrared (FTIR) spectroscopy (Nicolet Avatar 370DTGS) (Thermo Fisher Scientific Inc., Waltham, MA, USA). The microscopic morphology and structural changes of *M. aeruginosa* before and after exposure to nZVI were examined using a high-resolution scanning electron microscope (Nava450 Zeiss FEI-F50, Oberkochen, Germany), as detailed in Section S2.

### 2.4. Non-Targeted LC-MS Metabolomics

#### 2.4.1. LC-MS Sample Preparation

Each experimental group included six parallel samples, and six samples were collected as biological replicates for subsequent biological analyses. At the end of the reaction period, 10 mL of algal suspension was centrifuged (12,000 rpm, 10 min); the supernatant was discarded, the pellet was transferred to a sterile 2 mL microcentrifuge tube, washed three times with pre-chilled 10 mL PBS buffer, and then rapidly stored at −80 °C for subsequent LC–MS-based metabolomics analysis. After thawing at room temperature, 100 μL of samples were placed into 1.5 mL EP tubes. A 400 μL of acetonitrile (1:1) solution containing internal standards (L-2-chlorophenylalanine) was added, vortexed for 30 s, and sonicated at 5 °C and 40 KHz for 30 min. Samples were then stored at −20 °C for 30 min and centrifuged at 13,000× *g* for 15 min. The supernatant was dried with nitrogen, redissolved in 120 μL of acetonitrile (1:1), extracted by cryosonication at 5 °C and 40 KHz for 5 min, and centrifuged at 4 °C and 13,000× *g* for 10 min. The supernatant was transferred to an injection vial for analysis. Chromatographic conditions included two mobile phases: (A) 95% water and 5% acetonitrile, and (B) 47.5% acetonitrile, 47.5% isopropanol, and 5% water, both with 0.1% formic acid.

#### 2.4.2. LC-MS Analysis

The sample injection volume was set at 3 μL, with a column temperature maintained at 40 °C. Mass spectrometry was performed in positive and negative ion modes, with quality control samples embedded at intervals of 5–15 samples.

#### 2.4.3. Data Processing and Analysis

Collected raw data were refined using Progenesis QI v3.0 software (Waters Corporation, Milford, MA, USA), including baseline correction, peak detection and integration, retention time adjustment, data normalization, and logarithmic transformation. All metabolomics data were processed and analyzed on the online analysis platform provided by Shanghai Majorbio Bio-Pharm Technology Co., Ltd., Shanghai, China (www.majorbio.com) (No. 3, Lane 3399, Kangxin Highway, Pudong New Area, Shanghai). On this platform, multivariate statistical analyses were conducted via parameterized settings. First, principal component analysis (PCA) was performed to reveal the major sources of variation in an unsupervised manner; next, partial least squares–discriminant analysis (PLS-DA) was applied to discriminate among treatment groups and identify key discriminatory variables; hierarchical clustering analysis (HCA) was then used to delineate clustering relationships and inherent hierarchical structures among samples. On this basis, metabolites with significant differences were screened and identified using Student’s *t*-test and orthogonal partial least squares–discriminant analysis (OPLS-DA), with thresholds of *p* < 0.05 and variable importance in projection (VIP) > 1.

## 3. Results and Discussion

### 3.1. Metabolomics Analysis

To elucidate the changes in key metabolites following the nZVI reaction, the LC-MS technique was employed to analyze the metabolism of *M. aeruginosa* before and after treatment, aiming to gain insights into the metabolic pathways and overall impact on the *M. aeruginosa* molecular network.

#### 3.1.1. Data Quality Control and Metabolomic Analysis

During chromatographic analysis, sample components were separated by the chromatographic column and entered the mass spectrometer detector, producing serialized mass spectral images that were quantitatively analyzed to form a total ion flux map (Figure S1). Quality control (QC) samples, as well as PCA and PLS-DA analyses, were conducted under both positive and negative ion modes, with the procedures detailed in Section S3. The PCA (Figure S2a,b) revealed a high aggregation of QC samples and a clear separation between the nZVI and control groups, indicating distinct biometabolic differences in *M. aeruginosa*. PLS-DA analysis (Figure S2c,d) identified metabolites contributing to inter-group differences, revealing significant changes in metabolite content and composition due to nZVI exposure.

#### 3.1.2. Identification of Differential Metabolites

**Figure 1:**
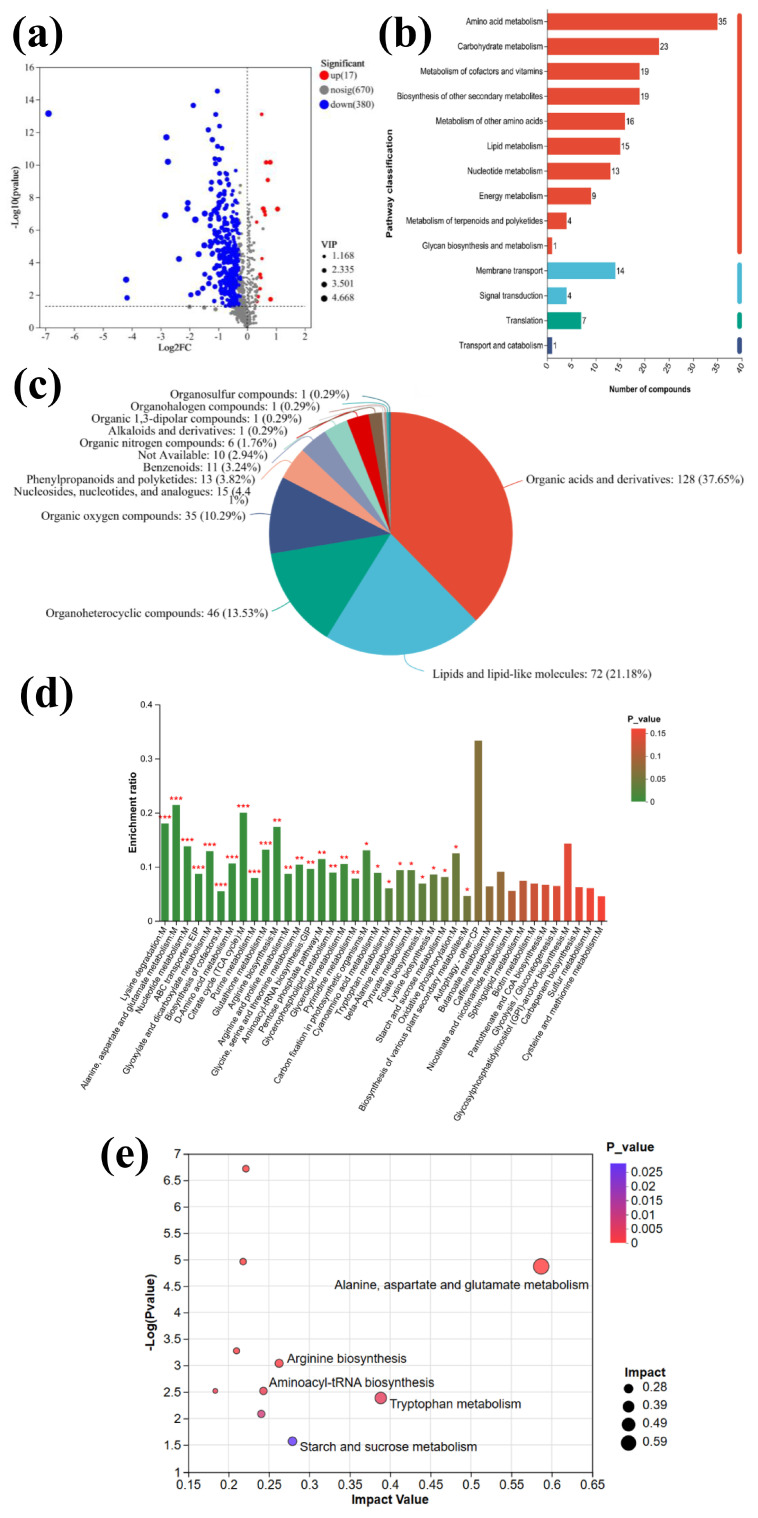
nZVI versus control: (**a**) volcano plot of differential metabolites; (**b**) classification plot of differential metabolites; (**c**) statistical plot of KEGG pathways of differential genes; (**d**) enrichment analysis of KEGG metabolic pathways; * *p* < 0.05, ** *p* < 0.01, *** *p* < 0.001; The color gradient of the bars indicates the significance of enrichment. By default, greener colors indicate that the KEGG term is more significantly enriched; and (**e**) analysis of KEGG bubble plots of significantly altered metabolites.

A volcano plot was utilized to visually comprehend the alterations in differential metabolites. As shown in Figure 1a, the horizontal coordinate represents the fold change in metabolite expression difference between the two groups (log_2_FC), and the vertical coordinate is the logarithm of the *p*-value. The horizontal dashed line represents *p* = 0.05, with points above and below indicating significant and non-significant metabolites, respectively [12]. Between the control and treatment groups, 397 significantly different metabolites were identified, including 17 up-regulated and 380 down-regulated metabolites. A systematic classification (Figure 1b) of the 397 differential metabolites revealed the top five categories: 128 organic acids and derivatives, 72 lipids and lipid molecules, 46 organic heterocyclic compounds, 35 organic oxygen compounds, and 15 nucleosides, nucleotides, and analogs. Differential metabolite appearance indicates that nZVI disrupted *M. aeruginosa* metabolism. nZVI had the greatest impact on lipid and organic acid metabolic synthesis, accounting for 37.65% and 21.18% of the metabolites, respectively, and more than 50% of total metabolic synthesis. The nZVI-treated and control groups were annotated and identified via online spectral matching on the Majorbio platform (www.majorbio.com). Table S1 lists the top twenty differential metabolites with significant VIP values. These metabolites generally showed a downward trend, with six having VIP values > 4, including five related to organic acids and one related to organic oxygen.

#### 3.1.3. Functional Pathway Analysis of Differential Metabolites

To deeply analyze nZVI’s inhibitory effect on *M. aeruginosa*, the significantly different metabolic pathways were examined. Figure 1c shows that genes with significant differential expression in the treatment group were involved in four primary pathways: metabolism, environmental information processing, genetic information conversion, and cellular processes. Metabolism-related pathways were notably activated, encompassing amino acid, lipid, cofactor enzymes and vitamin metabolism, carbohydrate metabolism, biosynthesis of secondary metabolites, nucleotide metabolism, energy metabolism, terpene and polyketide metabolism, and glycan biosynthesis. Lipid metabolism is crucial for the structural integrity of algae cells, as lipids are the main cell membrane components and play a defensive role against external intrusion [13]. Impaired lipid metabolism negatively impacts algae cell structure. In terms of lipid metabolism [14], 15 metabolites were identified and categorized in relevant pathways, revealing nZVI’s impact on lipid metabolism in *M. aeruginosa*. Among the 15 lipid metabolites, 3-β-D-galactosyl-sn-glycerol was up-regulated, possessing anti-inflammatory and antioxidant activities [15], while 14 lipid metabolites, including L-serine and 20-hydroxy leukotriene E4, were down-regulated. The down-regulation of most important lipid metabolism metabolites disrupted algae cell internal balance, leading to lipid peroxidation, antioxidant enzyme inactivation, and ultimately algae cell damage or death [16].

#### 3.1.4. Analysis of Differential Metabolite Enrichment

Kyoto Encyclopedia of Genes and Genomes (KEGG) is a comprehensive bioinformatics resource that focuses on metabolic pathways, gene functions, and the construction of biological system networks. In untargeted metabolomics analyses, KEGG provides standardized annotations and hierarchical classifications for metabolites, genes, and pathways, helping researchers map detected metabolites to known biological pathways and functional modules. Its pathway maps and associated annotation/enrichment tools support metabolite annotation, pathway enrichment analysis, and metabolic network reconstruction, making it a key platform for interpreting the biological significance of metabolomics data. Using the complete metabolite ensemble of *M. aeruginosa* as the enrichment background, the enrichment degree of metabolic pathways after nZVI treatment was deeply analyzed. By setting a significance threshold of *p* < 0.05, pathways with significant changes were selected. The enrichment rate directly reflects the impact on corresponding metabolic pathways, with higher rates indicating more prominent regulatory roles [17]. Figure 1d shows 28 enriched metabolic pathways in the treatment group, with 10 highly significantly enriched (*p* < 0.001), including lysine degradation, alanine, aspartate, and glutamate metabolism, nucleotide metabolism, ABC transport, glyoxylate, and dicarboxylic acid metabolism, cofactor biosynthesis, D-amino acid metabolism, citrate cycle, purine metabolism, and glutathione metabolism.

The metabolic pathway bubble diagram (Figure 1e) illustrates the results of the KEGG pathway analysis. Each bubble represents a distinct KEGG pathway, with the *x*-axis reflecting relative centrality and the *y*-axis indicating enrichment significance. The larger the bubble, the greater the importance of the pathway [18]. Among these pathways, the metabolic routes of alanine, aspartate, and glutamate are significantly enriched (Figure S3), and the expression patterns of nine metabolites are downregulated. These metabolites include L-glutamate, which plays a critical role in cellular metabolism and antioxidant reactions; aspartate, essential for amino acid metabolism, protein synthesis, and antioxidant responses; and N-acetyl-L-glutamate, vital for influencing amino acid and nitrogen metabolism, as well as regulating redox balance and metabolic homeostasis in algae cells. These findings indicate that the addition of nZVI significantly disrupts the cellular metabolism and redox state of *M. aeruginosa*, thereby hindering the synthesis of proteins, peptides, and nitrogen-containing nutrients.

#### 3.1.5. Analysis of Key Metabolites

Following nZVI treatment, carbohydrate metabolism was notably enhanced, with essential metabolites such as glucose 6-phosphate, ribulose 5-phosphate, and alginate 6-phosphate up-regulated, not only augments energy metabolism but also furnishes critical carbon backbones for other metabolic pathways [19]. Conversely, the TCA cycle (Figure S4), which is indispensable for the integration of carbohydrate, lipid, and protein metabolism, exhibited a decline in citric and malic acid concentrations. This diminution implies that algal cells may be reallocating energy to contend with external stress, thereby compromising the energy and material exchange mechanisms essential for the growth and metabolic processes of *M. aeruginosa* [20].

**Figure 2:**
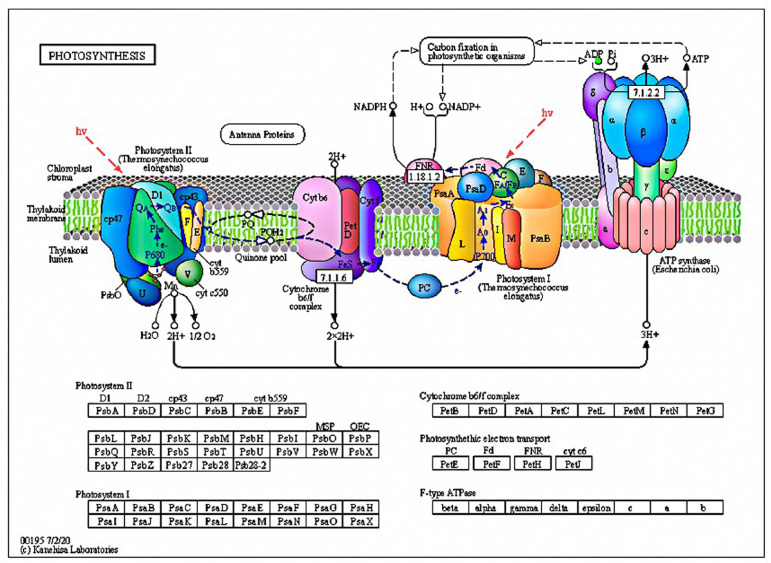
Photosynthetic pathways of *M. aeruginosa* in nZVI treatment group (green dots indicate down-regulation of metabolite epistasis factors).

Within plant chloroplasts, the Adenosine triphosphate (ATP) synthesized during the light reaction phase is crucial for the dark reaction and energy production. ATP, a fundamental indicator of cellular metabolism (Figure 2), is predominantly synthesized within the chloroplasts during photosynthesis and is largely sequestered there [21]. The coupling of photosynthetic and oxidative phosphorylation (Figure S5) processes interlinks ATP synthesis with electron transport. However, under the duress induced by nZVI treatment, the down-regulation of ADP, a molecule indispensable for the dark reaction, signifies an inhibition of the photosynthetic process in *M. aeruginosa*. This inhibition implies that nZVI treatment disrupts energy production and photosynthesis, further undermining the physiological vitality of *M. aeruginosa*.

#### 3.1.6. Metabolite Cluster Analysis

**Figure 3:**
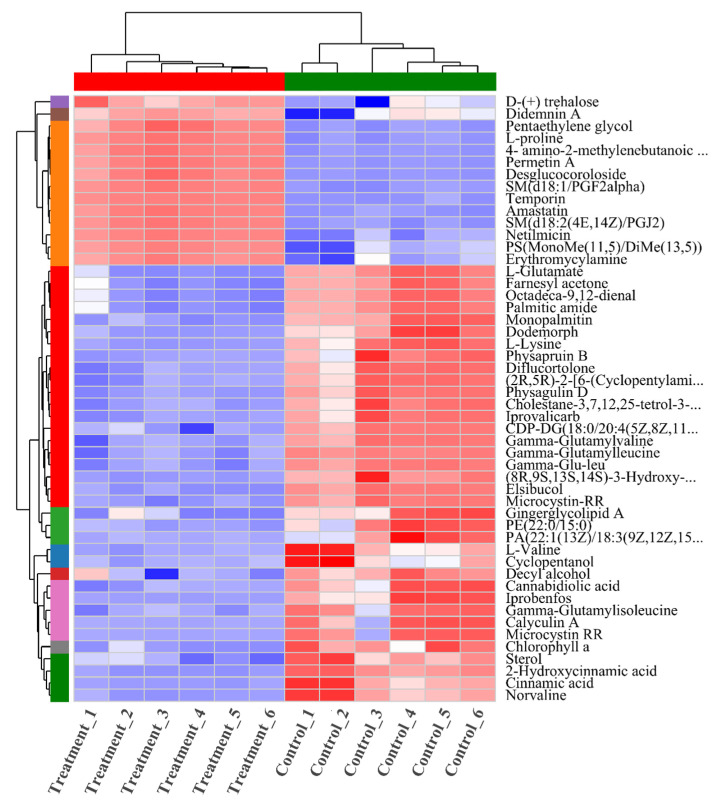
A clustered heatmap: each column represents a sample group, and each row represents a metabolite. The colors in the image indicate the relative expression levels of metabolites (red: high expression; blue: low expression). (Names that are too long have been truncated).

The preceding analysis indicates that, following nZVI treatment, significant differences in the extracellular metabolome of *M. aeruginosa* are mainly concentrated in lipids/lipid-like molecules and organic acids and their derivatives. To better visualize key signaling molecules released under stress, the top 50 metabolites by abundance were selected for hierarchical clustering analysis. The *x*-axis denotes individual samples and the *y*-axis denotes specific metabolites; the color scale reflects relative abundance, with blue indicating lower levels and red indicating higher levels. The dendrogram on the left displays metabolite clustering, with metabolite names on the right; shorter branch lengths indicate more similar abundance levels. The dendrogram at the top shows sample clustering, with sample names at the bottom; shorter branch lengths indicate greater similarity in overall metabolite expression profiles, more similar trends in abundance changes. Clustered heatmap analysis of the top 50 metabolite abundances revealed significant changes in the treatment group compared to the control group (Figure 3). This variation in metabolite patterns suggests that nZVI profoundly impacts the metabolic processes of *M. aeruginosa*. The results demonstrate that nZVI treatment promoted the accumulation of the amino acid metabolite L-proline, which is crucial for the formation of cell wall proteins, osmoregulation, and mitigation of both abiotic and biotic stresses in plants. The increased synthesis of L-proline implies that nZVI damages cell wall proteins, necessitating repair [22].

Furthermore, the upregulation of 4-amino-2-methylenebutanoic acid indicates a disruption in nitrogen metabolism, providing energy in response to stress. D-(+)trehalose, a key metabolite in the transformation processes of pentose and glucuronic acid [23], accumulates as nZVI impairs photosynthetic efficiency and inhibits carbohydrate transport within chloroplasts. The disruption of amino acid and carbohydrate metabolism is a primary cause of elevated ROS levels in algae cells. The downregulation of L-glutamate is primarily linked to the biosynthesis of N-formyl-methionyl-tRNA [24], likely due to a reduced tRNA synthesis rate in *M. aeruginosa* cells, thereby affecting protein synthesis and inhibiting algae growth. L-lysine, an essential amino acid for cellular processes, negatively regulates ROS production. After nZVI treatment, L-lysine synthesis in *M. aeruginosa* was inhibited, suggesting that the algae cells might be experiencing continuous oxidative stress [25]. Overall, these findings indicate that the treatment group significantly disrupted the metabolic processes related to oxidative stress and cell membrane integrity, affecting amino acids, carbohydrates, and pentoses.

### 3.2. Inhibitory Performance of nZVI on M. aeruginosa

#### 3.2.1. Changes in the Functional Groups of *M. aeruginosa*

**Figure 4:**
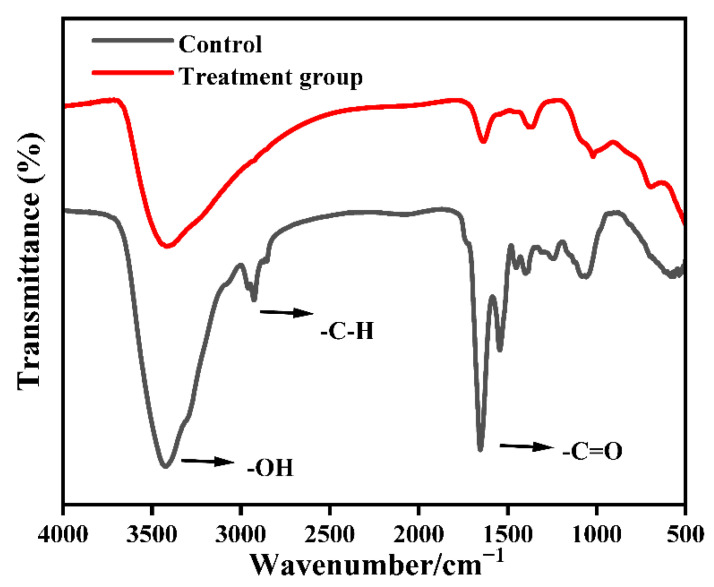
FTIR spectra of *M. aeruginosa* before and after nZVI treatment.

Spectral scans were performed on the constituents of *M. aeruginosa* cells from the two groups, and the results are presented in Figure 4. The absorption peak at 3420 cm^−1^ is typically associated with the stretching vibrations of hydroxyl (O-H) groups, indicating alterations in the integrity of the microalgae cell wall and changes in water content. The saturated C-H stretching vibrations in the 2926 cm^−1^ region, indicate the presence of abundant -CH_3_ and -CH_2_ functional groups within the compounds [26]. At the broad peak around 1655 cm^−1^, the absorption peak represented the stretching of protein C=O in the amide (C=O) I band, with a weakened intensity of its stretching vibrations. Near 1545 cm^−1^, the absorption peak was associated with the amide (C=O) II band, and after treatment, this peak became less pronounced, signifying the bending vibrations of N-H and stretching vibrations of C-N in proteins. This change might be due to varying degrees of disruption in the C-H bonds of proteins within the algae cells. Peng [27] et al. also observed similar phenomena, noting that the decrease in the intensity of the 1078 cm^−1^ peak was closely linked to DNA damage. Thus, this study postulates that nZVI treatment leads to a reduction in the peak intensities of -OH, -C-H, and -C=O functional groups, suggesting that the structure of the algae cell membrane has been compromised.

#### 3.2.2. Changes in Cell Morphology of Microalgae Cells

**Figure 5:**
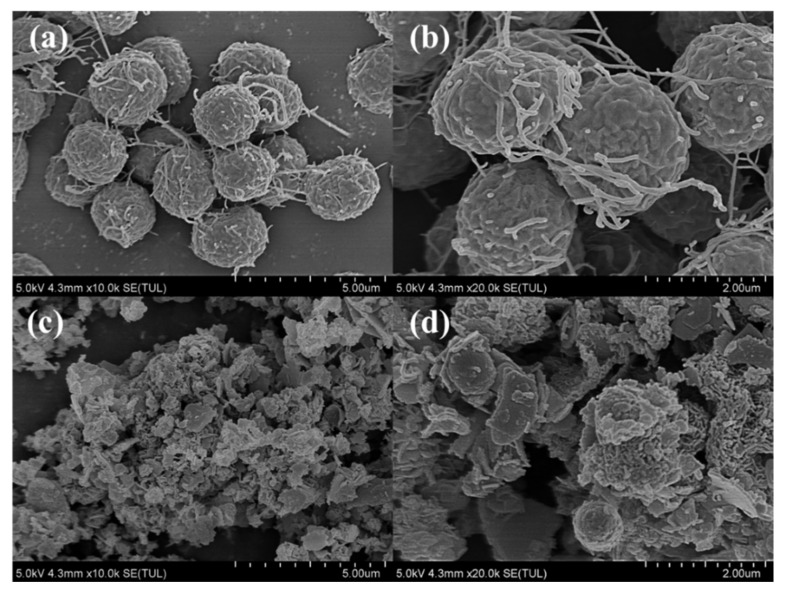
SEM images of morphological structures of algae cells (**a**,**b**) in control group and (**c**,**d**) in nZVI experimental group.

To further elucidate the inhibitory performance of nZVI on *M. aeruginosa*, SEM to observe the microstructure of cells before and after treatment. As shown in Figure 5a,b, before exposure, *M. aeruginosa* cells were spherical, with intact surfaces and clearly defined boundaries. These cells were structurally complete, and demonstrated a vigorous growth trend. In contrast, algae cells exposed to nZVI (Figure 5c,d) were enveloped by a layer of iron nanoparticles, which adhered firmly to the cell surface, leading to irregular cell morphology and rapid disintegration of the cell membrane until the cellular structure was severely compromised. Once inside the cell, the iron nanoparticles induced a series of oxidative damages, progressively impairing the cell membrane and organelles, resulting in extensive cell lysis and solute release.

## 4. Conclusions

In this study, we demonstrated the feasibility of using nano zero-valent iron (nZVI) to inhibit the growth of Microcystis aeruginosa. Untargeted metabolomics showed that nZVI markedly perturbs lipid and amino acid metabolism, suppresses protein synthesis, and impacts key pathways—including the TCA cycle, photosynthesis, and carbohydrate metabolism—thereby inducing imbalances in energy metabolism and overall metabolic homeostasis. FTIR spectra exhibited decreased intensities of -OH, -C-H, and C=O functional group peaks, indicating disruption of membrane components and structure. SEM observations revealed irregular cell morphology and membrane damage; once internalized, iron nanoparticles triggered successive oxidative injuries that progressively compromised membranes and organelles, ultimately leading to cell lysis and solute release. These findings provide insights to guide metabolomics studies of *M. aeruginosa* using nZVI.

## 5. Future Perspectives

This study centered on metabolomics to investigate the effects of nZVI on the metabolism of *M. aeruginosa*. To further validate and refine the underlying mechanisms in subsequent work, we will prioritize assessing cell viability and membrane integrity via live/dead staining coupled with confocal microscopy; employing cryo-scanning electron microscopy to minimize preparation-induced morphological artifacts; and supplementing chlorophyll fluorescence metrics (Fv/Fm, NPQ) and/or direct measurements of photosynthetic oxygen evolution to substantiate functional inhibition.

## Data Availability

The original contributions presented in this study are included in the article. Further inquiries can be directed to the corresponding authors.

## Supplementary Materials

The following supporting information can be downloaded at: https://www.mdpi.com/article/10.3390/toxics13110915/s1, Figure S1. Total ion chromatogram of QC sample in (a) positive ion mode and (b) negative ion mode. Figure S2. PCA (a,b) and PLS-DA (c,d) of *M. aeruginosa* metabolites in positive and negative ion mode after nZVI treatment. Figure S3. KEGG pathway for alanine, aspartate, and glutamate metabolism. Figure S4. Diagram of TCA cycling pathways in *M. aeruginosa* in nZVI treatment group. Figure S5. Oxidative phosphorylation pathways in *M. aeruginosa* in nZVI treatment group. Table S1. Differential metabolites between nZVI-treated and control groups (top 20). Figure S6. Effectiveness of nZVI in removing *M. aeruginosa*.
