# Supplementary material for: Sustainable Cyanobacterial Bloom Control: Inhibitory Effects of Nano Zero-Valent Iron on Microcystis aeruginosa and Metabolic Disruption
Source: Toxics. 2025 Oct 24;13(11):915. doi: 10.3390/toxics13110915 (PMC12656124; doi:10.3390/toxics13110915)
Supplement: Supplementary file 1 [file toxics-13-00915-s001.zip › toxics-3897707-supplementary.pdf]

Supplementary material

Sustainable Cyanobacterial Bloom Control: Inhibitory Effects of Nano Zero-Valent Iron on *Microcystis aeruginosa* and Metabolic Disruption

Guoming Zeng [1,2,3], Zilong Ma [3], Xiaoling Lei [1], Yong Xiao [2], Da Sun [4,5,*] and Yuanyuan Huang [1,2,*]

[1] Chongqing Academy of Science and Technology, Chongqing 401123, China
[2] School of Civil Engineering, Chongqing Jiaotong University, Chongqing 400074, China
[3] School of Civil and Hydraulic Engineering, Chongqing University of Science and Technology, Chongqing 401331, China
[4] Zhejiang Provincial Key Laboratory of Water Ecological Environment Treatment and Resource Protection, College of Life and Environmental Science, Wenzhou University, Wenzhou 325035, China
[5] Institute of Life Sciences and Biomedical Collaborative Innovation Center of Zhejiang Province, Wenzhou University, Wenzhou 325035, China
* Correspondence: sunday@wzu.edu.cn (D.S.); 18983093831@163.com (Y.H.)

Section S1. Preparation of nZVI

Five grams of $FeSO_4·7H_2O$ was weighed and added to 100 mL of a solution consisting of 70 mL deionized water and 30 mL anhydrous ethanol, followed by sonication for 15 min in a three-necked flask. Nitrogen gas was then introduced to expel the air, and the solution was stirred at 300 rpm. Subsequently, 2.04 grams of $NaBH_4$ was dissolved in 0.1% NaOH solution to prevent hydrolysis and added dropwise at a rate of one drop per second. Throughout the stirring process, nitrogen was continuously passed to prevent oxidation of the nZVI. After the addition, stirring continued with nitrogen gas for 20 min. The resulting mixture was washed 3 to 4 times with anhydrous ethanol and deionized water, and the supernatant was removed using solid-liquid separation by a strong magnetic method. The resulting black solid was dried at 60°C for 6 hours in a vacuum drying oven, then sealed and stored as nZVI.

Section S2. Characterization of algae cells

S2.1. Scanning electron microscope analysis

The algae cells were initially isolated through centrifugation at 4000 r/min for 20 min. Subsequently, the cells were fixed in a 2-3% glutaraldehyde solution at 4°C for two hours. After fixation, the cells underwent several rinsing cycles, 3 to 4 times, using a phosphate buffer solution (PBS) at pH 7.0 to ensure thorough washing. The cells were then dehydrated in a series of ethanol solutions with concentration gradients ranging from 30% to 100%. Finally, the samples were freeze-dried at -20°C overnight and observed under a field emission scanning electron microscope (Nava450 Zeiss FEI-F50, Germany).

S2.2. Fourier transform infrared (FTIR) spectroscopy analysis

The Microcystis aeruginosa suspension was lyophilized with a vacuum freeze dryer to obtain algal powder. The powder was then pressed into KBr pellets, and infrared absorbance spectra were recorded using a Fourier transform infrared (FTIR) spectrometer over the 400–4000 cm^{-1} range.

Section S3. Metabolomics Analysis

S3.1. Data Quality Control

During chromatographic analysis, the sample components were efficiently separated by the column before entering the mass spectrometer detector in sequence. The mass spectrometer performed successive scans on each effluent component, producing a series of mass spectral images. These images were quantitatively analyzed by summing the intensities of all ion signals to generate a single total ion current value. Using these data, a total ion current chromatogram was plotted, with time on the horizontal axis and total ion current on the vertical axis.

S3.2. PCA and PLS-DA Analysis of Metabolites

PCA was employed to observe the overall differences and variability of the samples within the group. Through dimensionality reduction and data simplification, each sample point exhibited distinct or aggregated patterns at the coordinate points on the graphs. The PCA score plots demonstrated a high degree of aggregation for QC samples with minimal dispersion, indicating a robust experimental process with excellent reproducibility. Under both positive and negative ion modes, the nZVI group showed significant separation from the control group on the PC1 axis, revealing differences in biometabolic characteristics. The marked separation on the PC2 axis suggested that nZVI induced a biological response in *M. aeruginosa*.

Data Quality Control

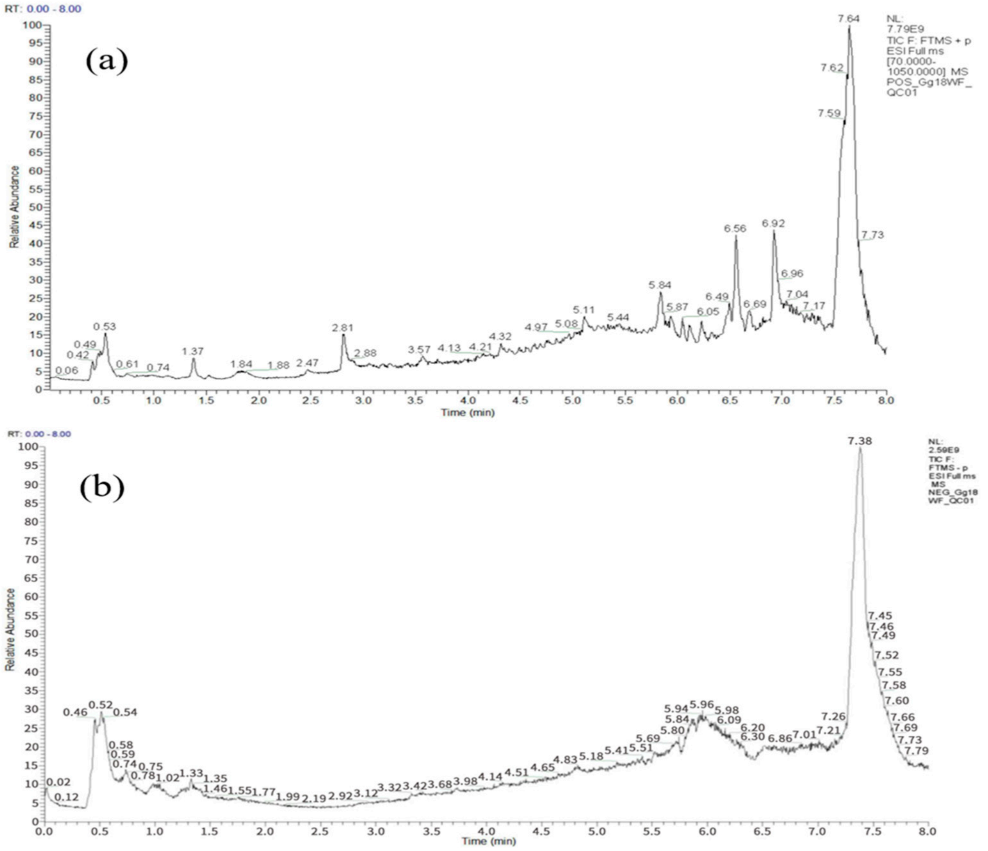

Figure S1. Total ion chromatogram of QC sample in (a) positive ion mode and (b) negative ion mode.

Analysis of Differential Metabolite Enrichment

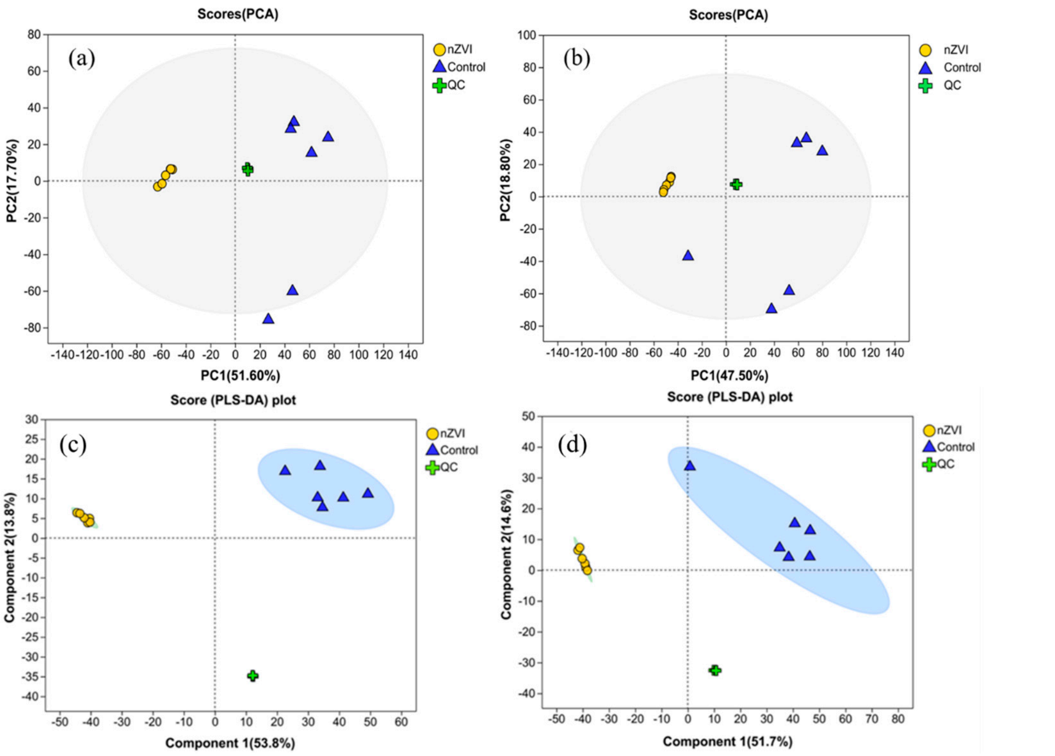

Figure S2. PCA (a-b) and PLS-DA (c-d) of *M. aeruginosa* metabolites in positive and negative ion mode after nZVI treatment.

Analysis of Differential Metabolite Enrichment

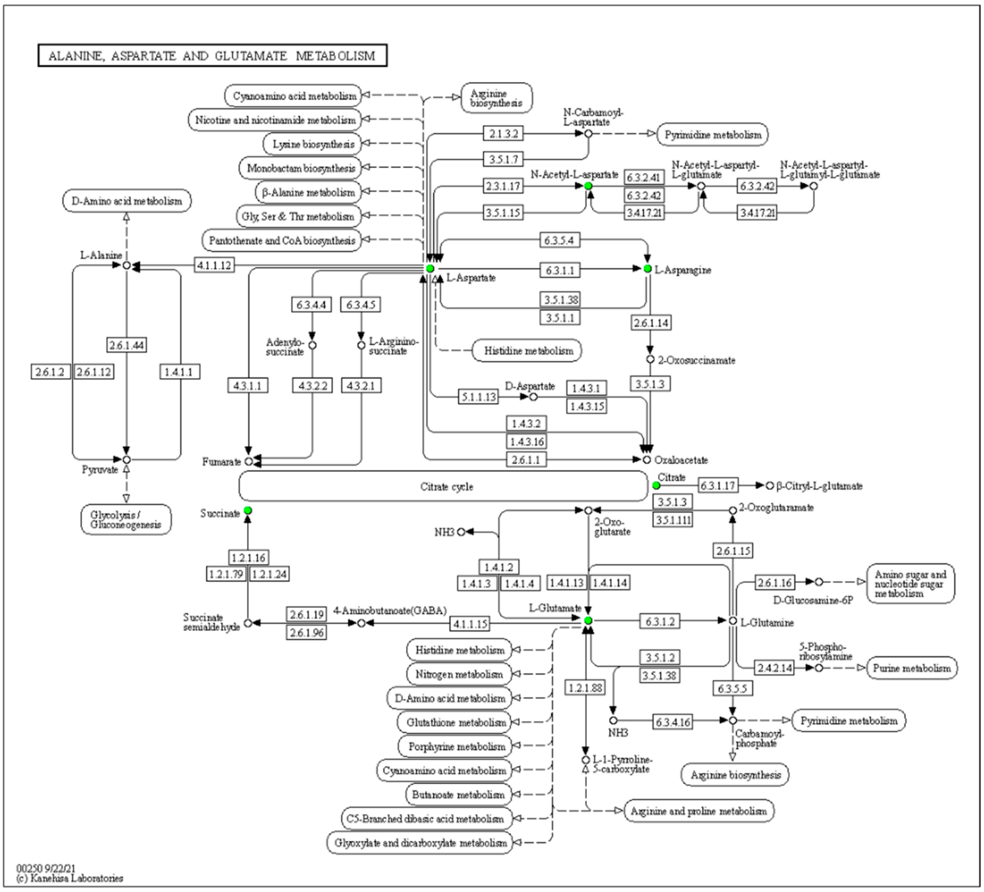

Figure S3. KEGG pathway for alanine, aspartate, and glutamate metabolism.

Analysis of Key Metabolites

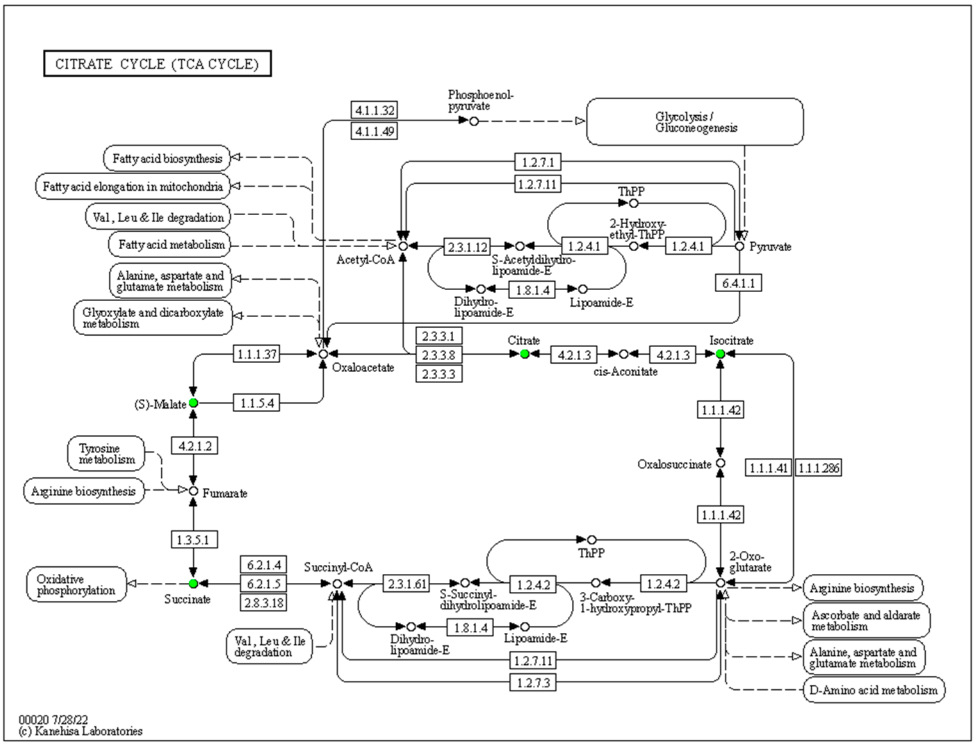

Figure S4. Diagram of TCA cycling pathways in *M. aeruginosa* in nZVI treatment group.

Analysis of Key Metabolites

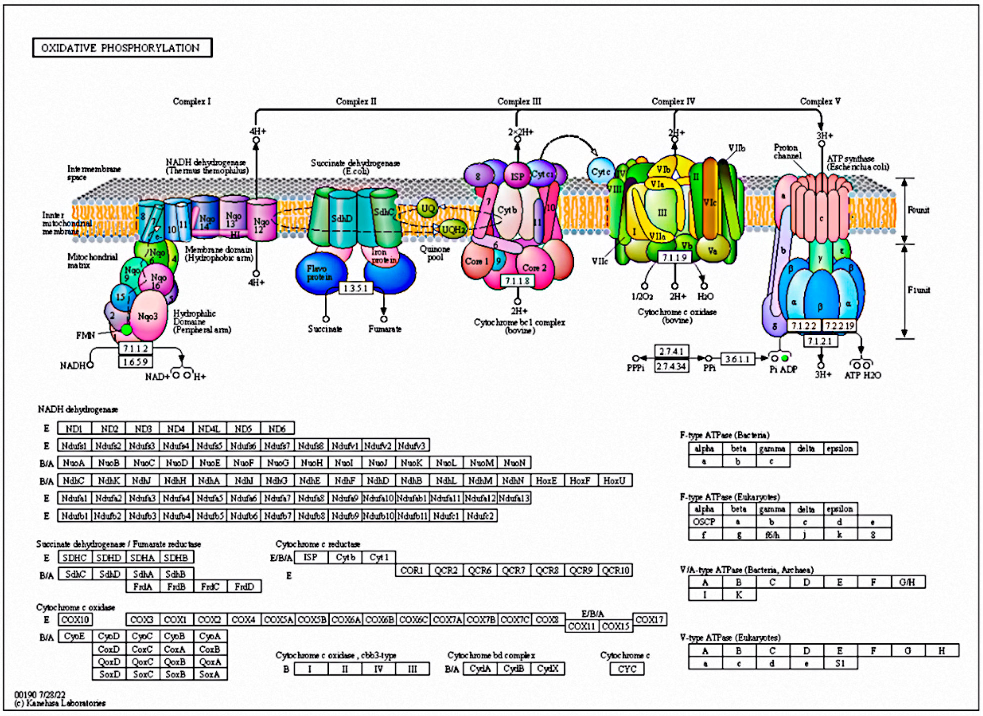

Figure S5. Oxidative phosphorylation pathways in *M. aeruginosa* in nZVI treatment group.

Table S1 Differential metabolites between nZVI-treated and control groups (top 20).

Metabolite name	Library ID	VIP	P	Expression Mode
Ile Glu Glu	-	4.6678	7.10E-14	down
Malic Acid	HMDB0000156; PW_C000592; MJDBOTE0000694	4.474	2.36E-07	down
Gamma-Glutamylproline	HMDB0029157	4.2932	1.29E-07	down
Ethyl glucuronide	HMDB0010325	4.1886	2.04E-12	down
L-Threo-3-Phenylserine	HMDB0002184	4.1222	6.49E-11	down
PE (16:1/0:0)	-	4.1153	0.001152	down
Isobutylidene	HMDB0302266	3.9294	8.91E-06	down
Pro Thr	-	3.7945	3.09E-05	down
3-(Methylthio)-1-propene	HMDB0031653	3.7893	9.95E-08	down
Geosmin	HMDB0036461	3.7493	6.12E-05	down
L-Glutamic gamma-semialdehyde	HMDB0002104; PW_C001415	3.5935	5.01E-08	down
11-alpha-O-beta-D-Glucopyranosyl-16alpha-O-methyl-neoquassin	HMDB0039773	3.474	0.00763	down
Tyrosyl-Lysine	HMDB0029110	3.4256	2.18E-08	down
Junosine	HMDB0038145	3.2376	6.64E-06	down
Citric Acid	PW_C000063; HMDB0000094; MJDBNL00000139	3.1959	6.63E-08	down
Gluten exorphin B5	HMDB0059795	3.133	0.003936	down
2-Amino-9-(4-amino-2-oxopyrimidin-1-yl)-1H-purin-6-one	HMDB0258125	3.1115	2.88E-12	down
3-Phosphoglycerate	HMDB0000807; HMDB0060180	3.1055	1.69E-06	down
D-Tartaric Acid	HMDB0029878; HMDB0000956; MJDBOTE0000633	3.0937	0.01504	down
N (6)-Methyllysine	HMDB0002038	3.054	2.24E-14	down

Section S4. Experimental evaluation of algal removal by nZVI

S4.1 Experimental methods

Algal suspensions without nZVI served as the control, and those with nZVI as the treatment group. Algal lysis experiments were conducted in 250 mL beakers (working volume 200 mL). The pH of the algal samples was first adjusted with 0.1 mol L^{-1} acetic acid and NaOH, after which nZVI was added to the respective groups, and the mixtures were stirred on a jar tester at a constant 300 rpm. At the end of the experiment, chlorophyll a (Chl-a) in the water samples was determined.

S4.2 Determination of chlorophyll a content

First, transfer 5 mL of algal suspension into a plastic centrifuge tube and centrifuge at 4000 rpm for 20 min, then carefully decant the clear supernatant. Add 5 mL of 80% acetone, mix thoroughly by shaking, disrupt the cells by ultrasonication, and centrifuge again at 4000 rpm for 15 min. Collect the clear supernatant for analysis, zero the spectrophotometer with 80% acetone as the reference, and measure absorbance at 653 nm and 666 nm. The chlorophyll a concentration is then calculated using the following equation:

$$\text{Chl-a}=15.65\times OD_{666}-7.34\times OD_{653} \quad (S4.1)$$

S4.3 Analysis of algal removal by nZVI

Under identical reaction conditions, the chlorophyll a content of algal cells exposed to nZVI was measured at 60 and 120 min. The results (Fig. S6) show that nZVI markedly suppressed the accumulation of chlorophyll a in Microcystis aeruginosa: at 60 min it decreased from 3.33 mg L-1 in the control to 0.76 mg/L, and at 120 min it decreased from 3.25 mg L^{-1} to 0.46 mg/L. These findings indicate that nZVI significantly reduces the chlorophyll a content of M. aeruginosa, thereby weakening photosynthesis and inhibiting algal growth.

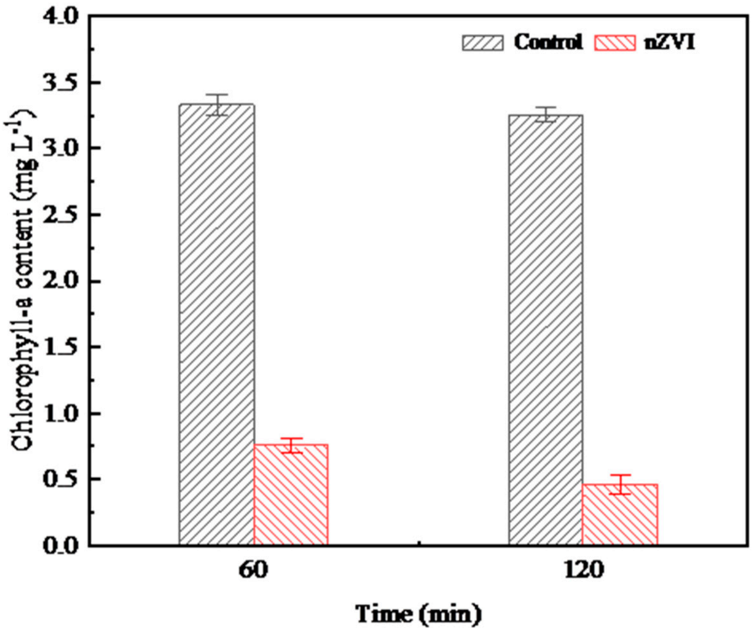

Figure S6. Effectiveness of nZVI in removing *M. aeruginosa.*
